# Cell phone addiction and sleep disturbance among medical students in Jiangsu Province, China: the mediating role of psychological resilience and the moderating role of gender

**DOI:** 10.3389/fpsyt.2024.1405139

**Published:** 2024-05-15

**Authors:** Bin Hu, Qi Wu, Yujia Xie, Liping Guo, Dehui Yin

**Affiliations:** Key Laboratory of Human Genetics and Environmental Medicine, School of Public Health, Xuzhou Medical University, Xuzhou, China

**Keywords:** psychological resilience, cell phone addiction, sleep disturbance, medical student, gender

## Abstract

**Background:**

Cell phone addiction presents a widespread and severe physical and mental health concern, now recognized as a global public health issue. Among medical college students, the issue of poor sleep quality has become particularly prevalent. This study aimed to investigate the relationship between cell phone addiction and sleep disturbance in a population of medical college students, exploring the potential mediating role of psychological resilience and the moderating impact of gender.

**Methods:**

A random cluster sampling method was employed to survey 5,048 students from four medical colleges in Jiangsu Province, China, utilizing the Mobile Phone Addiction Index (MPAI), Connor-Davidson Resilience Scale (CD-RISC), and Pittsburgh Sleep Quality Index (PSQI) for data collection. Statistical analyses were conducted using SPSS 26.0 and the PROCESS macro version 4.1. To assess mediation, Model 4 of the PROCESS macro was utilized, while Model 15 was employed to investigate the moderating effect of gender.

**Results:**

The results revealed a significant positive correlation between cell phone addiction and sleep disturbance, with psychological resilience found to partially mediate this relationship. Moreover, gender was observed to significantly moderate the impact of cell phone addiction on sleep disturbance. Specifically, bootstrap analysis indicated a significant interaction between cell phone addiction and gender (*Coeff.* = -0.0215, *P*< 0.001), with a stronger relationship found in males (*simple slope* = 0.0616, t = 16.66, *P*< 0.001) compared to females (*simple slope* = 0.0401, t = 9.51, *P<* 0.001).

**Conclusion:**

Ultimately, psychological resilience was identified as a partial mediator between cell phone addiction and sleep disturbance in medical students from Jiangsu Province, with gender playing a significant moderating role in this association.

## Introduction

College students (1), particularly medical students (2), are confronted with escalating academic and social pressures that often result in substandard sleep quality. Research shows that poor sleep quality is a common issue among medical students globally (3–5), with females reporting poorer sleep (6, 7). With the advancement of science and technology, cell phones have become indispensable in our daily lives. Although these devices offer convenience for various tasks like online learning, socializing, and entertainment, excessive use can lead to issues like sleep disorders and mental health problems (8, 9), significantly impacting overall well-being of college students. Studies have established a strong link between symptoms of cell phone addiction and the adverse effects it has on physical and mental health, particularly within the college student population (10, 11). Additionally, research has shown a direct association between cell phone addiction and mental health issues, as well as the quality of sleep (12–14).

Young people exhibiting addictive behaviors often experience emotional dysregulation, impaired cognitive abilities, and mental overload (15). The use of cell phone can compromise privacy, resulting in social isolation and negative feedback. Managing harmful emotions and behaviors, along with dealing with disconnection from the phone, can be distressing (16). Poor sleep quality observed in medical college students with addictive behaviors and negative emotions raises concerns (3, 17). Psychological resilience, which acts as a shield against adversity, plays a pivotal role in preventing negative behaviors like depression and cell phone addiction (18). Existing research has shown a positive relationship between psychological resilience and sleep quality (19–21). However, the impact of psychological resilience on the adverse effects of cell phone addiction on sleep disturbance remains unclear.

Previous studies show gender disparities in sleep quality and cell phone usage among youth, with females exhibiting higher levels of excessive cell phone use (22), which is closely associated with poorer sleep quality (23). Studies conducted at Yale University suggest that females may be more susceptible to stress-related psychological effects, potentially making them more vulnerable (24). The I-PACE model posits that individual traits, including gender, can impact problematic behaviors, yet the role of gender in cell phone addiction effects remains unresolved (25). This study delves into the intermediary function of psychological resilience in the relationship between cell phone addiction and sleep disturbance among medical college students, while also exploring whether gender plays a moderating role in this association.

**Figure 1:**
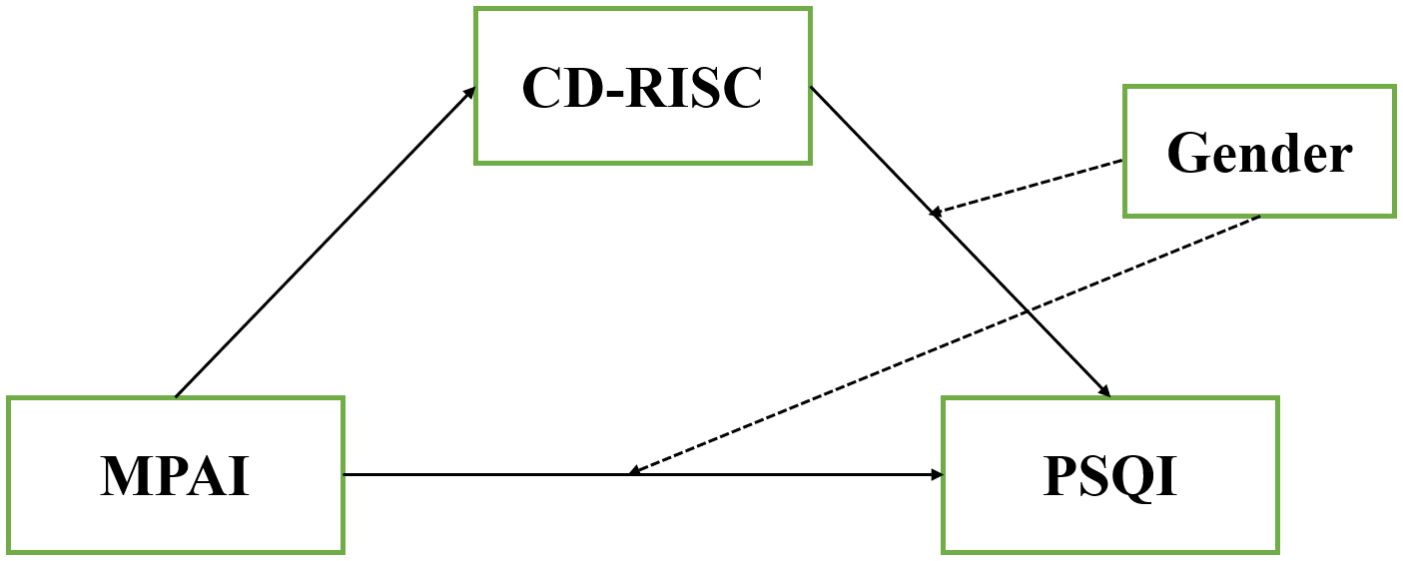
Conceptual research model.

The study was conducted among medical college students in Jiangsu Province, China, aiming to determine whether preventing cell phone addiction could enhance sleep quality among medical college students and to further understand the mechanisms through which psychological resilience and gender influence the adverse effects of cell phone addiction on sleep quality. The conceptual model is shown in **Figure 1**.

## Methods

### Participants

From October 27 to November 27, 2022, an online questionnaire survey was conducted on students from four medical colleges in Jiangsu Province, China, using a random cluster sampling method. A total of 5,048 participants were included in the final analysis, with 2540 (50.32%) being male and 2508 (49.68%) female. The questionnaire gathered general personal details including gender, grade, major, family economic level, frequency of physical activity, only-child status, and academic stress.

### Measures

#### Pittsburgh sleep quality index

The PSQI is used to assess the quality of sleep (26). It is a self-report scale consisting of 19 items (16 questions scored separately, excluding on bedtime and wake-up time) designed to collect a person's subjective perception of their sleep habits over a month. It assesses seven aspects of sleep quality, including “Subjective sleep quality”, “Sleep latency”, “Sleep duration”, “Habitual sleep efficiency”, “Sleep disturbance”, “Use of sleep medication”, and “daytime dysfunction”. Each item is scored from 0 to 3, with a total score ranging from 0 to 21, higher scores indicating more sleep disturbance. In this study, the Cronbach's alpha for the PSQI was 0.850 and the Kaiser-Meyer-Olkin value was 0.910.

#### Mobile phone addiction index

The mobile phone addiction index (MPAI) , revised by Leung (27), consists of 17 items scored from 1 to 5, assessing cell phone addiction among college students. It includes dimensions like “Inability to control craving”, “Anxiety and Feeling Lost”, “Withdrawal and escape” and “Productivity loss”. The higher total score, the greater level of cell phone addiction. In this study, the Cronbach's alpha for the MPAI was 0.918 and the Kaiser-Meyer-Olkin value was 0.903.

#### Connor-Davidson resilience scale

The Connor-Davidson Resilience Scale (CD-RISC) (28) is used to measure the ability to cope with stress and adversity. This study used a revised version (29) translated by Nan Xiao to measure the level of psychological resilience in college students. This scale contains 25 question entries divided into three dimensions of “Optimism”, “Self-improvement” and “Tenacity”, scored on a 5-point Likert scale: not true at all (0) to true almost all the time (4). The total score ranges from 0 to 100, with a higher total score indicating greater psychological resilience. In this study, the Cronbach's alpha for the CD-RISC was 0.968 and the Kaiser-Meyer-Olkin value was 0.975.

### Statistical analysis

In order to mitigate the potential impact of common method biases, the study employed Harman's single-factor test. This approach entailed conducting an exploratory factor analysis on a combined set of items from three scales, totaling 60 items. The analysis is designed to identify the number of factors with eigenvalues greater than 1. To assess the presence of common method bias, the researchers compared the percentage of variance explained by the first factor against a threshold of 40%. If the variance explained by the first factor exceeded 40%, it indicated a significant common method bias; conversely, if it fell below 40%, it suggested minimal bias.

Different statistical analyses, including descriptive statistical analysis, t-test and Pearson's correlation analysis, were performed using IBM SPSS 26.0. Prior to analysis, all measurements were tested for normality to ensure they followed a normal distribution. The data presentation in the tables incorporated the use of N (%) for categorical variables and mean ± standard deviation (SD) for numerical variables. Pearson's correlation analysis was used to examine the relationship between cell phone addiction, psychological resilience and sleep disturbance. Model 4 (30) of PROCESS macro 4.1 of SPSS 26.0 was used to examine the mediating effect of psychological resilience, while Model 15 (31) was used to explore the moderating role of gender.

Model 4 is used to explore the mediation role of psychological resilience in the association between cell phone addiction and sleep disturbance. This involves analyzing the effect of cell phone addiction on psychological resilience, followed by an evaluation of how psychological resilience influenced sleep disturbance. The significance of the mediation effect was assessed by calculating the product of these effects and constructing confidence intervals for this product. A significant mediation effect was indicated by confidence intervals that did not include zero.

Model 15 was used to investigate the moderating role of gender on both the direct and indirect effects of cell phone addiction on sleep disturbance through psychological resilience. This model accounted for the direct impact of cell phone addiction on sleep disturbance, while also examining how psychological resilience acted as a mediator this relationship. Importantly, interaction terms were introduced to analyze how gender moderated the direct effect (Cell Phone Addiction x Gender interaction) as well as the mediating effect through psychological resilience (Psychological Resilience x Gender interaction). The hypothesis model is shown in **Figure 1**.

## Results

### Common method biases

The Harman's single-factor test was used to conduct exploratory factor analysis on 60 items across 3 scales. Of the total of 8 factors identified, those with an eigenvalue exceeding 1 were considered. Notably, the variance explained by the first factor was 27.399%, falling short of the critical criterion of 40% threshold, indicating an absence of substantial common method bias in this study.

### Characterization analysis

Regarding participant demographics, information encompassed gender, grade, native place, only child or not, family economy, BMI, smoking status, drinking status, and physical exercise frequency. Notably, 59.1% of the participants hailed from urban areas, with a majority not being only (57.8%). Refer to **Table 1** for a detailed analysis.

**Table 1:** Characterization of the participants for the full samples and by gender (n = 5048).

Variables	All samples	Male	Female	$X^{2}$	
	n (%)	n (%)	n (%)		
Grade				33.58	<0.001
First year	3047 (60.3)	1629 (64.2)	1418 (56.6)		
Second year	1316 (26.1)	618 (24.3)	698 (27.8)		
Third year or more	685 (13.6)	293 (11.5)	392 (15.6)		
Native place				5.502	0.019
Rural	2063 (40.9)	1079 (42.5)	984 (39.2)		
Urban	2985 (59.1)	1461 (57.5)	1524 (60.8)		
Only child or not				83.97	<0.001
Yes	2129 (42.2)	1232 (48.5)	897 (35.8)		
No	2919 (57.8)	1308 (51.5)	1611 (64.2)		
Family Economy				7.81	0.02
High	421 (8.3)	209 (8.2)	212 (8.4)		
Middle	3928 (77.8)	1945 (76.6)	1983 (79.1)		
Low	699 (13.9)	386 (15.2)	313 (12.5)		
BMI				202.45	<0.001
<18.5	839 (16.6)	315 (12.4)	524 (20.9)		
18.5-24	3052 (60.5)	1444 (56.9)	1608 (64.1)		
>24	1157 (22.9)	781 (30.7)	376 (15.0)		
Smoking				145.40	0.000
Never	4708 (93.3)	2262 (89.0)	2446 (97.5)		
Occasional	244 (4.8)	195 (7.7)	49 (2.0)		
Usually	96 (1.9)	83 (3.3)	13 (0.5)		
Drinking				265.30	<0.001
Never	3219 (63.8)	1344 (52.9)	1875 (74.8)		
Occasional	1757 (34.8)	1140 (44.9)	617 (24.6)		
Usually	72 (1.4)	56 (2.2)	16 (0.6)		
Physical exercise				140.18	<0.001
≤1 per month	823 (16.3)	302 (11.9)	521 (20.8)		
1-3times per week	3260 (64.6)	1615 (63.6)	1645 (65.6)		
4-7times per week	965 (19.1)	623 (24.5)	342 (13.6)		

The overall mean MPAI score was 43.79 ± 13.84, while the mean CD-RISC score averaged at 61.72 ± 19.01, and the PSQI score at 5.15 ± 2.87. The primary focus of our inquiry pertained to gender difference, with descriptive analyses indicating that females registered significantly higher mean scores on both the MPAI and PSQI scales compared to males. Moreover, female scores were higher across all dimensions of the MPAI and PSQI scales, except for Sleep latency, Sleep duration and Use of sleep medication. The total CD-RISC score and its dimensions were significantly lower in females than in males (*P*< 0.05). Detailed information is shown in **Table 2**.

**Table 2:** t-test comparisons of clinical variables between male and female participants.

Variables	Full examples	Male	Female	*P*
	$\bar{x}\pm s$	$\bar{x}\pm s$	$\bar{x}\pm s$	
MPAI				
Total	43.79±13.84	42.34±14.48	45.25±13.00	<0.001
Inability to control craving	16.58±5.59	16.36±5.90	16.80±5.25	0.005
Anxiety and Feeling Lost	8.67±3.25	8.17±3.31	9.18±3.12	<0.001
Withdrawal and escape	9.81±4.25	9.46±4.28	10.16±4.19	<0.001
Productivity loss	8.73±3.11	8.35±3.19	9.11±2.97	<0.001
CD-RISC				
Total	61.72±19.01	62.79±21.02	60.63±16.66	<0.001
Optimism	9.46±3.21	9.58±3.50	9.33±2.89	0.006
Self-improvement	20.89±6.30	21.11±6.98	20.68±5.52	0.016
Tenacity	31.37±10.40	32.11±11.36	30.62±9.27	<0.001
PSQI				
Total	5.15±2.87	5.00±3.03	5.33±2.70	<0.001
Subjective sleep quality	0.90±0.66	0.88±0.69	0.92±0.62	0.032
Sleep latency	0.82±0.84	0.82±0.85	0.82±0.83	0.864
Sleep duration	0.75±0.67	0.79±0.70	0.72±0.63	0.001
Habitual sleep efficiency	0.77±1.14	0.70±1.10	0.85±1.18	<0.001
Sleep disturbance	0.95±0.66	0.91±0.70	0.99±0.61	<0.001
Use of sleep medication	0.12±0.46	0.16±0.51	0.08±0.40	<0.001
Daytime disfunction	0.97±0.80	0.95±0.82	0.98±0.78	<0.001

### Correlation analysis

Controlling for gender, grade, BMI, native place and being only child or not, partial correlation analysis was used. Findings revealed a negative correlation between the total MPAI score and the total CD-RISC score (r=-0.099, *P*<0.001), alongside positive correlation with the total PSQI score (r=0.273, *P*<0.001) and gender (r=0.105, *P*<0.001). Furthermore, the total CD-RISC score was negatively correlated with total PSQI score (r=-0.203, *P*<0.001), gender (r=-0.057, *P*<0.001), native place (r=-0.079, *P*<0.001), only child or not (r=-0.056, *P*<0.001). Total PSQI scores were positively correlated with gender (r=0.061, *P*<0.001), grade (r=0.111, *P*<0.001), native place (r=0.046, *P*<0.01) and only child or not (r=0.047, *P*<0.01), while negatively correlating with BMI (r=-0.036, *P*<0.05) (**Table 3**).

**Table 3:** Pearson's correlations between the variables.

Variables	1	2	3	4	5	6	7	8
1.MPAI	1.000							
2.CDRISC	-0.099^***^	1.000						
3.PSQI	0.273^***^	-0.203^***^	1.000					
4.Gender	0.105^***^	-0.057^***^	0.061^***^	1.000				
5.Grade	0.012	-0.028	0.111^***^	0.081^***^	1.000			
6.Native place	0.025	-0.079^***^	0.046^**^	0.033^*^	-0.005	1.000		
7.Only child or not	0.019	-0.056^***^	0.047^**^	0.129^***^	-0.002	0.325^***^	1.000	
8.BMI	-0.015	0.013	-0.036^*^	-0.194^***^	-0.005	-0.017	-0.053^***^	1.000

*P<0.05; **P<0.01; ***P<0.001.

### Mediation effect analysis

Model 4 in SPSS Macro PROCESS 4.1 was used to examine the mediating effects. Controlling for grade, BMI, native place, and being only child or not, cell phone addiction was significantly negatively associated with psychological resilience (*β*= -0.132, *P*< 0.001). Cell phone addiction was found to be significantly positively associated with sleep disorder (*β*= 0.053, *P*< 0.001). Psychological resilience was significantly negatively associated with sleep disorder (*β*= -0.026, *P*< 0.001).

**Figure 2:**
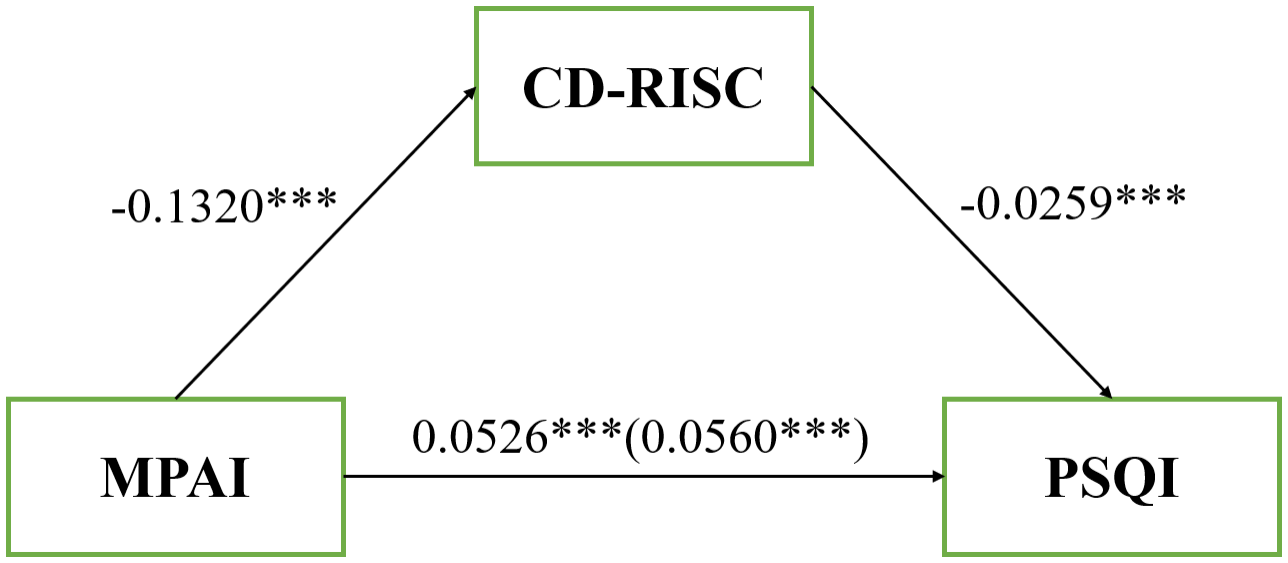
Effect of cell phone addition on sleep quality with the mediation of psychological resilience ***P< 0.001.

The results derived from the 5,000 bootstrap samples showed that all indirect effects were statistically significant, with the 95% confidence interval excluding 0. The total effect of cell phone addiction on sleep disturbance was estimated at 0.0560 (*P*< 0.001). The indirect effect of psychological resilience was scaled as 0.0034, with a 95% CI (0.0019,0.0052), accounting for 6.07% of the total effect (0.0034/0.0560). This analysis suggests that psychological resilience partially function as a mediator in the relationship between cell phone addiction and sleep disorder (**Table 4** and **Figure 2**).

**Table 4:** Mediating effect test of psychological flexibility between cell phone addiction and sleep quality among college students.

	CD-RISC				PSQI			
			Bootstrap 5000 Times 95%CI				Bootstrap 5000 Times 95%CI	
	*β*	S.E.	LLCI	ULCI	*β*	S.E.	LLCI	ULCI
Constant	74.113^***^	1.736	70.710	77.517	3.684^***^	0.290	3.116	4.253
MPAI	-0.132^***^	0.019	-0.170	-0.094	0.053^***^	0.003	0.047	0.058
CD-RISC	–	–	–	–	-0.026^***^	0.003	-0.030	-0.022
Grade	-0.708	0.368	-1.429	0.014	0.412^***^	0.053	0.309	0.516
Native place	-2.557^***^	0.571	-3.676	-1.438	0.101	0.082	-0.060	0.262
Child or not	-1.257^*^	0.569	-2.372	-1.142	0.149	0.082	-0.010	0.309
BMI	0.253	0.425	-0.580	1.085	-0.127^*^	0.061	-0.247	-0.008
	R^2^=0.017				R^2^=0.119			
	F=17.889				F=112.896			

*P<0.05; **P<0.01; ***P<0.001.

### Moderated mediation effect analysis

**Figure 3:**
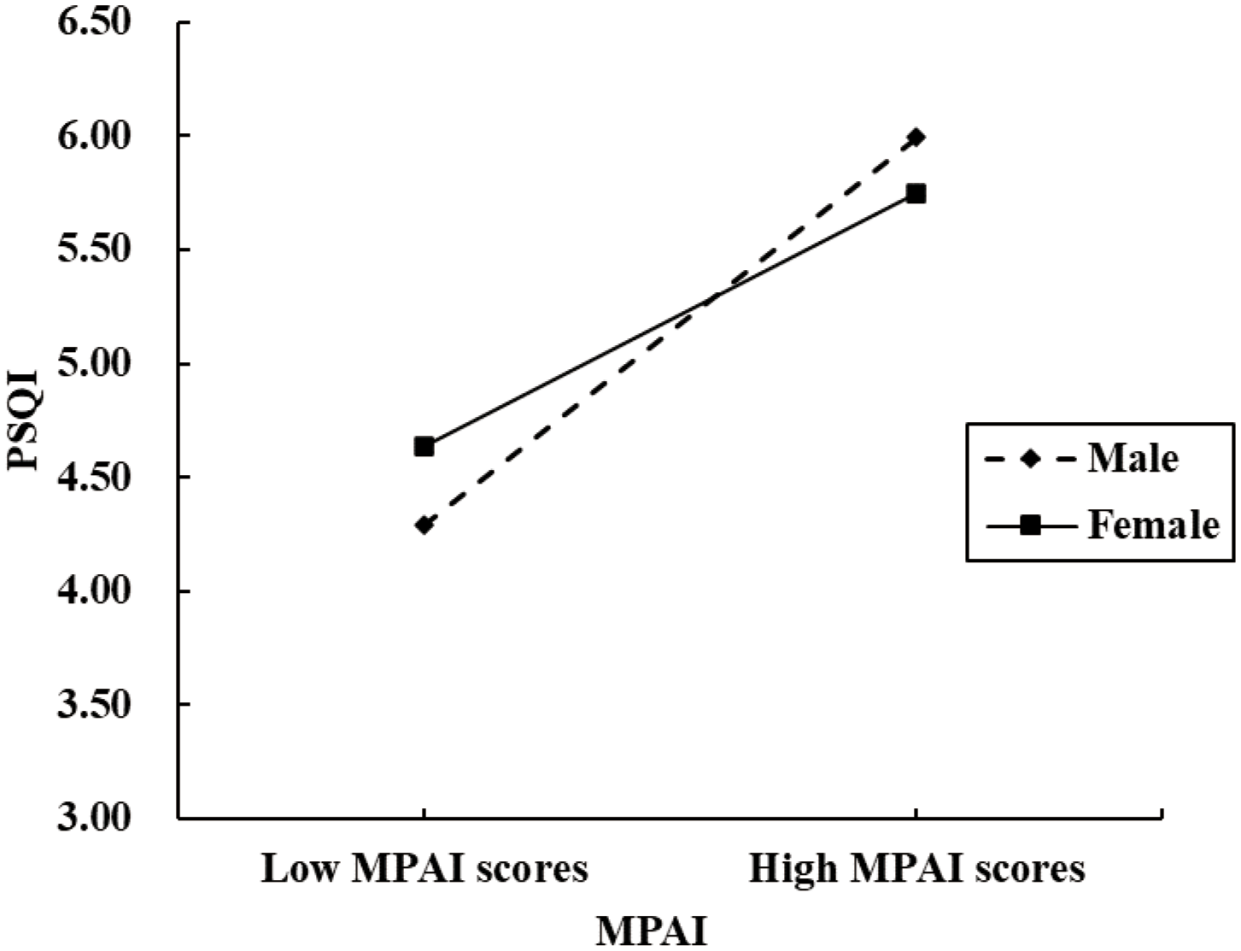
The moderating role of gender in MPAI and PSQI.

After controlling for grade, BMI, native place, and being only child or not, Model 15 of the SPSS Macro PROCESS was applied to evaluate the proposed moderating mediator model, with psychological resilience as the mediator and gender as the moderator. Specifically, gender was incorporated as a dummy variable (male = 0, female = 1). The results showed that gender moderated the direct path but not the second half of the mediated path, as shown in **Table 5**. Bootstrap test results showed that the interaction term between MPAI and gender significantly predicted PSQI (*Coeff.*= -0.0215, *P*<0.001), indicating that gender plays a moderating role in the relationship between MPAI and PSQI. Further simple slope analysis was performed, as shown in **Figure 3**. The level of cell phone dependence was positively correlated with the sleep disturbance in both males (*simple slope* = 0.0616, t = 16.66, *P*< 0 .001) and females (*simple slope* = 0.0401, t = 9.51, *P*< 0 .001), revealing that as cell phone dependence increased, sleep disturbance worsened for both genders.

**Table 5:** Tests the moderated mediation effect of cell phone addiction on Sleep quality.

Moderator W(Gender)	Mediating variable M(CD-RISC)		Dependent variable Y(PSQI)	
	*Coeff.*	t	*Coeff.*	t
X: MPAI	-0.132	-6.8813^***^	0.0509	18.185^***^
W: Gender			0.0499	0.6344
X×W			-0.0215	-3.8409^***^
M: CD-RISC			-0.0271	-13.0271^***^
M×W			-0.0059	-1.4168
R^2^	0.0174		0.1212	
ΔR^2^			0.0026	
			(ΔF(5048)=14.75, *P*<0.001)	
Conditional indirect effect for different gender values (1=male,2=female)				
Gender	Effect	BootSE.	Bootstrap 5000 Times 95%CI	
			LLCI	ULCI
1(-0.4968)	0.0032	0.0009	0.0017	0.005
2(0.5032)	0.004	0.001	0.0021	0.0061
Moderated mediation index	0.0008	0.0007	-0.0005	0.0022

***P<0.001.

## Discussion

In this study, the total scores for sleep quality and cell phone addiction were significantly higher among female medical students compared to male medical students. Conversely, male medical students scored significantly higher than their female counterparts on the total score for psychological resilience. Our analysis of a mediation model and a moderating mediation model revealed that psychological resilience plays a crucial role in mitigating the adverse effects of cell phone addiction on sleep quality. Moreover, the research indicated that female medical students are more likely to experience poorer sleep quality due to cell phone addiction.

Our findings align with previous reports indicating that medical college students with higher levels of cell phone addiction tend to experience poorer sleep quality (14, 32). In the medical college student population, cell phone addiction can affect an individual's emotional self-control, emotional balance, and potentially leading to negative emotions such as anxiety or depression, all of which can impact sleep quality. Furthermore, the emission of blue light from cell phone screens inhibits melatonin secretion, ultimately contributing to reduced sleep quality from a physiological standpoint (33).

Psychological resilience partially mediates the relationship between cell phone addiction and poor sleep quality among medical college students. Individuals with higher levels of cell phone addiction may quickly recognize negative emotions, such as anxiety and loneliness, and have lower levels of psychological resilience. Conversely those with high psychological resilience do not routinely contemplate all potential stress-related implications in their daily lives, thereby experiencing better sleep compared to their counterparts with lower levels of psychological resilience (34).

The moderating mediator model suggests that gender moderates the relationship between cell phone addiction and sleep disturbance. Specifically, female medical students with cell phone addiction are more prone to experiencing poorer sleep quality. Data analysis revealed that female medical students exhibited lower psychological resilience scoresand higher cell phone addiction scores than their male counterparts. Although women reported better sleep quality in the study by Adams SK et al, their habit of answering phone calls before sleep may lead to mood fluctuations or sleep disturbances (35). We suggest that in comparison to male counterparts, female medical students might engage more in self-presentation through mobile social media to seek approval from others. This cognitive load of strategizing for approval, coupled with lower psychological resilience, could render female medical students more susceptible to sleep disturbances stemming from bedtime cognitive arousal (36).

Our study focuses on cell phone addiction and sleep disturbance among medical students within the context of a larger phenomenon. Cell phone addiction is closely linked to internet and social media addiction, which can impact various daily activities, including sleep. It is essential to acknowledge this intricate relationship to fully grasp its effect on sleep health. In conclusion, our results align with the "I-PACE" model, suggesting that disorders stemming from addictive behaviors result from interactions between core individual traits and various moderating and mediating factors (25).

Based on the findings of this study, it is recommended that educational institutions, families, and society prioritize addressing sleep disturbances among medical students by establishing proper support systems for sleep disorders. Furthermore, due to the adverse emotional consequences of mobile phone addiction, educational institutions should raise awareness about the potential harms of such addictions and their relationship with sleep disorders. They should guide medical students, particularly females, in developing a balanced approach to the use of cell phones and the internet to reduce addictive behaviors and promote emotional well-being, thus enhancing psychological resilience. Providing a conducive environment, interventions such as positive thinking training can be particularly beneficial for students experiencing sleep issues (37). Software developers could also assist by creating programs that remind users of their phone usage time or offer regular feedback on their usage habits to aid in understanding and managing phone addiction.

Our research emphasizes the significant impact of psychological resilience, cell phone addiction, and gender on the sleep patterns of medical students. We propose practical preventive measures and innovative technological solutions to enhance sleep quality and address cell phone addiction. Additionally, our study underscores the importance of fostering psychological resilience through targeted positive interventions. The findings advocate for a comprehensive approach combining educational strategies and technological advancements to enhance the quality of sleep among students. Such interventions not only benefit individual well-being but also positively influence medical students' academic performance and career preparedness.

### Limitations and prospects

The current study has several limitations that need to be addressed. Firstly, the study only gathered questionnaires from medical college students in Jiangsu Province, China. To enhance the study's scope and generalizability, future research should consider expanding the survey to include a more diverse population. Secondly, the survey's reliance on personal subjective assessments may compromise the objectivity of the study results. Subsequent studies could benefit from incorporating objective assessment methods like clinician diagnosis and polysomnography (PSG) examination. Thirdly, the study did not thoroughly examine various sleep patterns such as onset latency or intermittent awakenings. To address this limitation, future research should conduct a comprehensive analysis of these aspects to deepen the understanding of factors affecting sleep quality in university students. Additionally, the online survey format hindered the accurate calculation of response rates, potentially biasing the sample toward individuals more inclined to complete the survey, thus affecting the generalizability of the findings, and we did not collect data on smoking status, alcohol and caffeine intake, and physical activity, all of which are important variables that may need to be adjusted. These factors could potentially have a significant impact on our study results. Despite these limitations, the results of this study remain significant. The findings reaffirm the pivotal role of cell phone addiction in predicting sleep disturbance among medical college students. Furthermore, the mediating effect analysis demonstrated that psychological resilience mitigated the impact of cell phone addiction on sleep disturbance. Additionally, the study elucidated the moderating role of gender as an individual characteristic in the relationship between cell phone addiction and sleep disturbance.

## Data availability statement

The raw data supporting the conclusions of this article will be made available by the authors, without undue reservation.

## Ethics statement

The studies involving humans were approved by The Ethics Committee of Xuzhou Medical University (approved number: xzhmu-2022079). The studies were conducted in accordance with the local legislation and institutional requirements. The participants provided their written informed consent to participate in this study.

## Author contributions

BH: Investigation, Methodology, Writing – original draft. QW: Data curation, Investigation, Writing – original draft. YX: Investigation, Writing – review & editing. LG: Investigation, Writing – review & editing. DY: Conceptualization, Writing – review & editing, Methodology.

## References

[1] Benham G Charak R Cano-Gonzalez I Mena Teran J Kenemore J. Recent stressful life events and perceived stress as serial mediators of the association between adverse childhood events and insomnia. Behav Med. (2024), 1–12. doi: 10.1080/08964289.2024.2335175 38634227

[2] Shafiee A Fili J Ghafari S Sattari MA Borna N Pourramzani A. The prevalence of sleep disturbance and its possible associated factors among Iranian medical students: A cross-sectional study with a national meta-analysis. Sleep Med X. (2024) 7:100107. doi: 10.1016/j.sleepx.2024.100107 38374870 PMC10875233

[3] Hammad MA Alyami MHF Awed HS. The association between internet addiction and sleep quality among medical students in Saudi Arabia. Ann Med. (2024) 56:2307502. doi: 10.1080/07853890.2024.2307502 38294763 PMC10833109

[4] Duthie CJ Cameron C Smith-Han K Beckert L Delpachitra S Garland SN. Sleep Management Strategies among Medical Students at the University of Otago. Behav Sleep Med. (2023) 21:448–59. doi: 10.1080/15402002.2022.2127723 36178287

[5] Souza AKR Sandes RS Vasco RFV Albuquerque EVA. Quality of sleep and excessive daytime sleepiness among medical students in a Brazilian private university. Rev Assoc Med Bras (1992). (2024) 70:e20231141. doi: 10.1590/1806-9282.20231141 38656002 PMC11042822

[6] Xie J Li X Luo H He L Bai Y Zheng F. Depressive symptoms, sleep quality and diet during the 2019 novel coronavirus epidemic in China: A survey of medical students. Front Public Health. (2020) 8:588578. doi: 10.3389/fpubh.2020.588578 33575239 PMC7870982

[7] Marelli S Castelnuovo A Somma A Castronovo V Mombelli S Bottoni D. Impact of Covid-19 lockdown on sleep quality in university students and administration staff. J Neurol. (2021) 268:8–15. doi: 10.1007/s00415-020-10056-6 32654065 PMC7353829

[8] Herrell C Foster S. Can't stop won't stop: Problematic phone use, sleep quality, and mental health in U.S. Graduate students. J Am Coll Health. (2024), 1–7. doi: 10.1080/07448481.2024.2334068 38546702

[9] Goel A Moinuddin A Tiwari R Sethi Y Suhail MK Mohan A. Effect of smartphone use on sleep in undergraduate medical students: A cross-sectional study. Healthcare (Basel). (2023) 11:2891. doi: 10.3390/healthcare11212891 37958035 PMC10649238

[10] Chen Z Xiong J Ma H Hu Y Bai J Wu H. Network analysis of depression and anxiety symptoms and their associations with mobile phone addiction among Chinese medical students during the late stage of the Covid-19 pandemic. SSM Popul Health. (2024) 25:101567. doi: 10.1016/j.ssmph.2023.101567 38524176 PMC10958643

[11] Abuhamdah SMA Naser AY. Smart phone addiction and its mental health risks among university students in Jordan: A cross-sectional study. BMC Psychiatry. (2023) 23:812. doi: 10.1186/s12888-023-05322-6 37936164 PMC10631016

[12] Feng Z Diao Y Ma H Liu M Long M Zhao S. Mobile phone addiction and depression among Chinese medical students: The mediating role of sleep quality and the moderating role of peer relationships. BMC Psychiatry. (2022) 22:567. doi: 10.1186/s12888-022-04183-9 35999533 PMC9396829

[13] Wang W Wu M Zhu Z Ma L Zhang L Li H. Associations of mobile phone addiction with suicide ideation and suicide attempt: Findings from six universities in China. Front Public Health. (2023) 11:1338045. doi: 10.3389/fpubh.2023.1338045 38312140 PMC10834704

[14] Nahidi M Ahmadi M Fayyazi Bordbar MR Morovatdar N Khadem-Rezayian M Abdolalizadeh A. The relationship between mobile phone addiction and depression, anxiety, and sleep quality in medical students. Int Clin Psychopharmacol. (2024) 39:70–81. doi: 10.1097/yic.0000000000000517 37781789

[15] Di Nicola M Ferri VR Moccia L Panaccione I Strangio AM Tedeschi D. Gender differences and psychopathological features associated with addictive behaviors in adolescents. Front Psychiatry. (2017) 8:256. doi: 10.3389/fpsyt.2017.00256 29249992 PMC5716988

[16] Hawk ST van den Eijnden RJJM van Lissa CJ ter Bogt TFM. Narcissistic adolescents' Attention-seeking following social rejection: links with social media disclosure, problematic social media use, and smartphone stress. Comput Hum Behav. (2019) 92:65–75. doi: 10.1016/j.chb.2018.10.032

[17] Garmabi M Andishmand Z Naderi F Sharifnezhad A Darrudi F Malekzadeh R. The prevalence of depression and anxiety and its association with sleep quality in the first-year medical science students. Depress Res Treat. (2024) 2024:7102081. doi: 10.1155/2024/7102081 38651016 PMC11035008

[18] Gong Z Lv Y Jiao X Liu J Sun Y Qu Q. The relationship between Covid-19-related restrictions and fear of missing out, problematic smartphone use, and mental health in college students: the moderated moderation effect of resilience and social support. Front Public Health. (2022) 10:986498. doi: 10.3389/fpubh.2022.986498 36203674 PMC9530251

[19] Arora T Grey I Östlundh L Alamoodi A Omar OM Hubert Lam KB. A systematic review and meta-analysis to assess the relationship between sleep duration/quality, mental toughness and resilience amongst healthy individuals. Sleep Med Rev. (2022) 62:101593. doi: 10.1016/j.smrv.2022.101593 35462348

[20] Lenzo V Sardella A Musetti A Freda MF Lemmo D Vegni E. The relationship between resilience and sleep quality during the second wave of the Covid-19 pandemic: A longitudinal study. Nat Sci Sleep. (2022) 14:41–51. doi: 10.2147/nss.S344042 35023980 PMC8747773

[21] Shi Y Bai Y Zhang L Chen Y Liu X Liu Y. Psychological resilience mediates the association of the middle frontal gyrus functional connectivity with sleep quality. Brain Imaging Behav. (2022) 16:2735–43. doi: 10.1007/s11682-022-00735-5 36307619

[22] González-Bueso V Santamaría JJ Fernández D Merino L Montero E Ribas J. Association between internet gaming disorder or pathological video-game use and comorbid psychopathology: A comprehensive review. Int J Environ Res Public Health. (2018) 15:668. doi: 10.3390/ijerph15040668 29614059 PMC5923710

[23] Claesdotter-Knutsson E André F Fridh M Delfin C Hakansson A Lindström M. Gender-based differences and associated factors surrounding excessive smartphone use among adolescents: Cross-sectional study. JMIR Pediatr Parent. (2021) 4:e30889. doi: 10.2196/30889 34813492 PMC8663478

[24] Lowe SR Hennein R Feingold JH Peccoralo LA Ripp JA Mazure CM. Are women less psychologically resilient than men? Background stressors underlying gender differences in reports of stress-related psychological sequelae. J Clin Psychiatry. (2021) 83:21br14098. doi: 10.4088/JCP.21br14098 34936244

[25] Brand M Young KS Laier C Wölfling K Potenza MN. Integrating psychological and neurobiological considerations regarding the development and maintenance of specific internet-use disorders: An interaction of person-affect-cognition-execution (I-pace) model. Neurosci Biobehav Rev. (2016) 71:252–66. doi: 10.1016/j.neubiorev.2016.08.033 27590829

[26] Becker SP Jarrett MA Luebbe AM Garner AA Burns GL Kofler MJ. Sleep in a large, multi-university sample of college students: Sleep problem prevalence, sex differences, and mental health correlates. Sleep Health. (2018) 4:174–81. doi: 10.1016/j.sleh.2018.01.001 PMC5863586 29555131

[27] Leung L. Linking psychological attributes to addiction and improper use of the mobile phone among adolescents in Hong Kong. J Children Media. (2008) 2:93–113. doi: 10.1080/17482790802078565

[28] Connor KM Davidson JR. Development of a new resilience scale: The connor-davidson resilience scale (Cd-risc). Depress Anxiety. (2003) 18:76–82. doi: 10.1002/da.10113 12964174

[29] Yu X Zhang J. Factor analysis and psychometric evaluation of the connor-davidson resilience scale (Cd-risc) with chinese people. Soc Behav Personality: an Int J. (2007) 35:19–30. doi: 10.2224/sbp.2007.35.1.19

[30] Fritz MS Mackinnon DP. Required sample size to detect the mediated effect. Psychol Sci. (2007) 18:233–9. doi: 10.1111/j.1467-9280.2007.01882.x PMC2843527 17444920

[31] Igartua JJ Hayes AF. Mediation, moderation, and conditional process analysis: Concepts, computations, and some common confusions. Spanish J Psychol. (2021) 24:e49. doi: 10.1017/SJP.2021.46 35923144

[32] Uzunçakmak T Ayaz-Alkaya S Akca A. Prevalence and predisposing factors of smartphone addiction, sleep quality and daytime sleepiness of nursing students: A cross-sectional design. Nurse Educ Pract. (2022) 65:103478. doi: 10.1016/j.nepr.2022.103478 36327595

[33] Selmaoui B Touitou Y. Association between mobile phone radiation exposure and the secretion of melatonin and cortisol, two markers of the circadian system: A review. Bioelectromagnetics. (2021) 42:5–17. doi: 10.1002/bem.22310 33238059

[34] Li Y Guo K. Research on the relationship between physical activity, sleep quality, psychological resilience, and social adaptation among chinese college students: A cross-sectional study. Front Psychol. (2023) 14:1104897. doi: 10.3389/fpsyg.2023.1104897 36844303 PMC9950505

[35] Adams SK Kisler TS. Sleep quality as a mediator between technology-related sleep quality, depression, and anxiety. Cyberpsychol Behav Soc Netw. (2013) 16:25–30. doi: 10.1089/cyber.2012.0157 23320870

[36] Almeida F Marques DR Gomes AA. A preliminary study on the association between social media at night and sleep quality: The relevance of fomo, cognitive pre-sleep arousal, and maladaptive cognitive emotion regulation. Scand J Psychol. (2023) 64:123–32. doi: 10.1111/sjop.12880 36256468

[37] Ding X Wang X Yang Z Tang R Tang YY. Relationship between trait mindfulness and sleep quality in college students: A conditional process model. Front Psychol. (2020) 11:576319. doi: 10.3389/fpsyg.2020.576319 33132983 PMC7550415

